# Combinatorial recognition of a complex telomere repeat sequence by the *Candida parapsilosis* Cdc13AB heterodimer

**DOI:** 10.1093/nar/gkv092

**Published:** 2015-02-08

**Authors:** Olga Steinberg-Neifach, Kemar Wellington, Leslie Vazquez, Neal F. Lue

**Affiliations:** 1 Department of Microbiology & Immunology, W. R. Hearst Microbiology Research Center, Weill Medical College of Cornell University, 1300 York Avenue, New York, NY 10065, USA; 2 Hostos Community College, City University of New York, 500 Grand Concourse, Bronx, NY 10451, USA

## Abstract

The telomere repeat units of *Candida* species are substantially longer and more complex than those in other organisms, raising interesting questions concerning the recognition mechanisms of telomere-binding proteins. Herein we characterized the properties of *Candida parapsilosis* Cdc13A and Cdc13B, two paralogs that are responsible for binding and protecting the telomere G-strand tails. We found that Cdc13A and Cdc13B can each form complexes with itself and a heterodimeric complex with each other. However, only the heterodimer exhibits high-affinity and sequence-specific binding to the telomere G-tail. EMSA and crosslinking analysis revealed a combinatorial mechanism of DNA recognition, which entails the A and B subunit making contacts to the 3′ and 5′ region of the repeat unit. While both the DBD and OB4 domain of Cdc13A can bind to the equivalent domain in Cdc13B, only the OB4 complex behaves as a stable heterodimer. The unstable Cdc13AB_DBD_ complex binds G-strand with greatly reduced affinity but the same sequence specificity. Thus the OB4 domains evidently contribute to binding by promoting dimerization of the DBDs. Our investigation reveals a rare example of combinatorial recognition of single-stranded DNA and offers insights into the co-evolution of telomere DNA and cognate binding proteins.

## INTRODUCTION

The ends of linear eukaryotic chromosomes, or telomeres, play critical roles in maintaining genome stability (1–3). In most organisms, telomere DNAs consist of copies of a short asymmetric sequence that is G-rich for the 3′-end-bearing strand (G-strand). Because the G-strand is typically longer than the complementary C-strand, most chromosomes terminate in 3′-overhangs commonly referred to as G-tails. Both the duplex region of telomeres and the G-tails are bound by protective proteins, and these proteins collectively block DNA repair factors from engaging in aberrant ‘repair’ of the natural chromosome ends as if they are double strand breaks.

**Figure 1.**
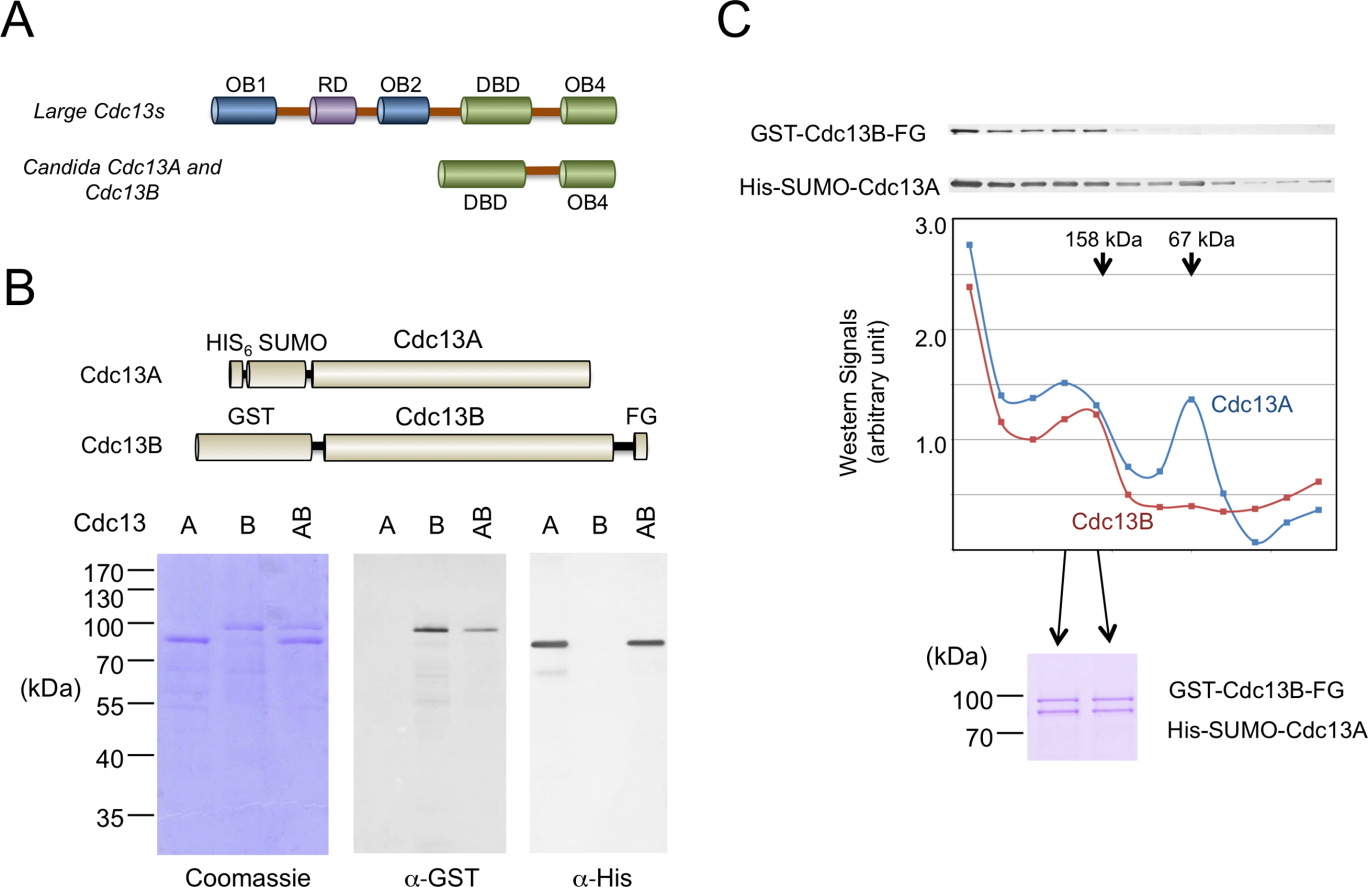
Cdc13 domain organizations and the purification of *Cp*Cdc13A, *Cp*Cdc13B and *Cp*Cdc13AB complexes. (**A**) The domain organizations of large Cdc13s and small Cdc13s (found mainly in *Candida* species) are schematically illustrated. (**B**) Top: The affinity-tagged Cdc13A and Cdc13B used for expression and purification in this study are illustrated schematically. Bottom: Separately expressed Cdc13A and Cdc13B were purified by Ni-NTA and glutathione-Sepharose chromatography, respectively. The Cdc13AB complex was purified from a strain co-expressing both paralogs by sequential Ni-NTA and FLAG affinity chromatography. All three preparations were analyzed by SDS-PAGE, Coomassie staining and Western. (**C**) Top: The Cdc13AB complex purified by Ni-NTA and FLAG affinity chromatography was fractionated through a glycerol gradient. The distributions of Cdc13A and Cdc13B in the fractions were analyzed by Western using α-His and α-GST antibodies, respectively. Middle: Signals from the western analyses were plotted. The arrows indicate the positions of the BSA (67 kDa) and aldolase (158 kDa) standards fractionated through a parallel gradient. Bottom: Two fractions that correspond to the Cdc13AB heterodimeric complex were analyzed by SDS-PAGE and Coomassie staining.

The telomere repeat units in the majority of organisms are quite short and regular (< or = 8 bp). Indeed, many organisms in diverse phyla (including fungi, protists, plants and metazoans) share a prototypical 6-bp repeat (5′-TTAGGG-3′/5′-CCCTAA-3′) that is bound by members of well-conserved protein families (4,5). Conspicuously different are fungi in the *Saccharomyces, Kluyveromyces* and *Candida* genera, which belong to the Saccharomycotina subphylum of budding yeast. The telomere repeat units of these organisms are extraordinarily divergent and differ from the typical repeats in being long (typically 12–25 bp), occasionally irregular, and having reduced G/C content (6,7). The emergence of such repeats and the co-evolution of telomere proteins in these organisms pose interesting evolutionary and mechanistic questions that remain to be addressed. One issue that has attracted considerable attention is the DNA recognition mechanisms of telomere proteins, i.e. the mechanisms by which the single strand (ss) and double strand (ds) telomere-binding proteins in these organisms recognize the complex and divergent target sites (8–13). Perhaps not surprisingly, the duplex telomere and G-tail binding proteins of Saccharomycotina yeast are distinct from those found in organisms with the prototypical repeat. In particular, the G-tails of the majority of organisms are bound by Pot1 homologues, whereas those of Saccharomycotina yeast by Cdc13 (14,15). Structurally, Cdc13 homologues display considerable plasticity, with the *Saccharomyces* and *Kluyveromyces* family members carrying four OB fold domains (henceforth referred to as large Cdc13s), and the *Candida* family members carrying just two OB folds (referred to as the small Cdc13s) (Figure 1A) (16). These domains mediate distinct functions in a modular fashion. In the ‘large’ Cdc13s such as *Sc*Cdc13, the OB1 domain is responsible for dimerization as well as binding to Pol1 (the catalytic subunit of DNA polymerase α) (17). The OB2 domain also forms dimers and may modulate interaction with Stn1, another telomere capping protein that functions together with Cdc13 (18). The last two domains, DBD (the DNA-binding domain and the third OB fold) and OB4 (the fourth and final OB fold), are responsible for high-affinity DNA binding and interaction with Stn1, respectively (17,19). Based on sequence alignments and functional characterizations, the ‘small’ Cdc13s contain just the DBD and OB4 domains, and utilize the former for DNA-binding and the latter for dimerization and Stn1-interaction (12,20). Surprisingly, recent analysis of *Candida* genomes revealed a second small Cdc13 that most likely arose through gene duplication. Genetic analysis of the two small Cdc13s (named Cdc13A and Cdc13B) in *Candida albicans* suggests that the two paralogs perform overlapping but non-redundant functions in telomere regulation (20).

We have sought to address the mechanistic and evolutionary issues related to the divergent *Candida* telomere repeats by surveying the DNA-binding mechanisms of Cdc13 homologues in multiple species. In one study, we found that the *C. albicans* (*Ca*) Cdc13A and B paralogs preferentially form heterodimeric complexes, although each protein can also self-associate to form homo-oligomers (20). Both the AA and AB complex (but not the BB complex) can bind with high affinity to the *C. albicans* telomere G-tail, although the sequence requirements for these interactions were not examined in detail. In a separate study, we more thoroughly analyzed the DNA binding properties of the *Candida tropicalis* Cdc13A alone, and found that high-affinity interaction requires two copies of a 6-nt sequence element (GGATGT) in the DNA substrate, as well as dimerization of Cdc13A through its OB4 domain (12). Because the 6-nt element is shared by many *Candida* telomeres (7), *Ct*Cdc13AA can bind several heterologous telomere repeats *in vitro*. For example, in DNA-binding assays, the formation of the complex between *Ct*Cdc13AA and a *C. tropicalis* G-strand probe is significantly inhibited by excess G-strand DNA competitors derived from *C. albicans, Candida orthopsilosis* and *Candida parapsilosis*, which all carry the 6-nt consensus sequence. These findings suggest that the AA dimer lacks stringent species-specificity, but leaves open the recognition mechanisms of the AB dimer. Here we report the characterization of the Cdc13s in a third *Candida* species, *C. parapsilosis (Cp)*. We found that like their homologues in *C. albicans, Cp*Cdc13A and *Cp*Cdc13B can each self-associate and form heterodimers with each other. However, only the AB complex exhibits high affinity for the *Cp* telomere G-tail. Surprisingly, high-affinity binding requires just one copy of the telomere repeat unit, and hence just one copy of the 6-nt element. Additional studies indicate that recognition of G-tail by *Cp*Cdc13AB is highly species-specific, and that the complex recognizes the cognate G-tail in a combinatorial fashion, with the DBDs of Cdc13A and Cdc13B contacting the 3′ and 5′ region of the telomere repeat unit, respectively. These results reveal unexpected complexity and species-specificity in the recognition mechanisms of *Candida* Cdc13s and suggest means by which different Cdc13s may have evolved highly tailored binding specificity for the cognate telomere repeat unit.

## MATERIALS AND METHODS

### Co-expression and extract preparation

The DNAs encoding full-length *Cp*Cdc13A and *Cp*Cdc13B, as well as various domains (see Table 1 for the amino acids included in each expression construct), were amplified by PCR and cloned into the pSMT3 vector (21) or the pGEX6P-1 vector (GE Healthcare) to enable their expression as HIS_6_-SUMO or GST fusion proteins, respectively. The pSMT3 vector was constructed by inserting the SUMO open reading frame in between the *Nhe*I and *Bam*HI sites of pET-28a. Some reverse primers used for PCR contain the FLAG tag to enable purification and detection of the fusion protein through the FLAG antibody. Each HIS_6_-SUMO fusion protein was expressed alone or co-expressed with a GST fusion protein in *Escherichia coli* BL21 (DE3). The growth and induction protocols as well as the extract preparation procedures were as previously described (12).

**Table 1.**
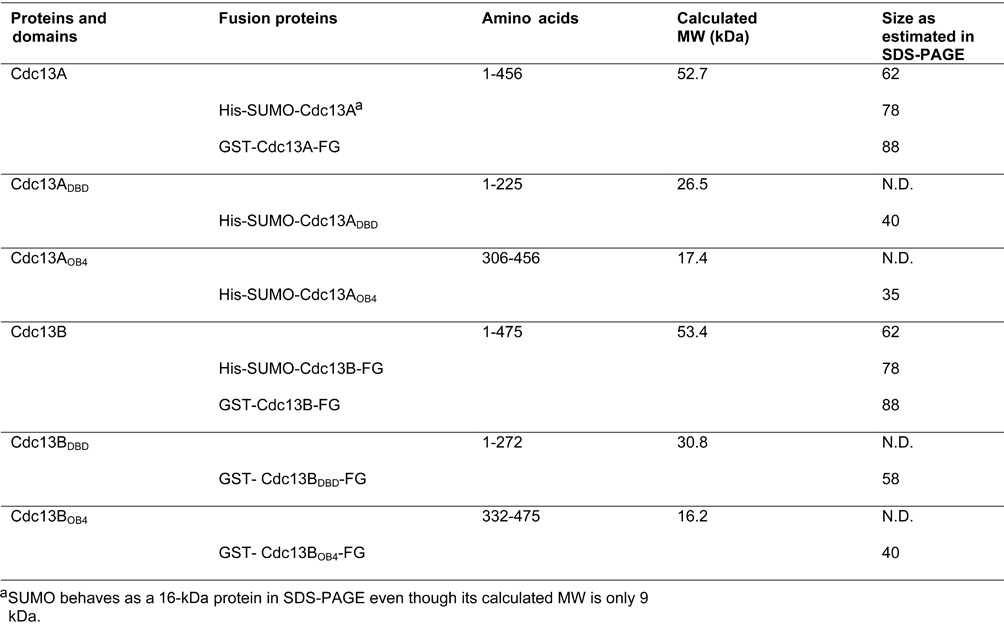
Proteins and protein domains used in this study

### Affinity purification and glycerol gradient fractionation

Proteins or protein complexes containing the His_6_-SUMO tag were purified over Ni-NTA columns as follows. Extracts were mixed with Ni-NTA resin (extract: resin = 10:1) at 4°C for 2 h with constant rotations. The suspension was poured into a 10-ml Poly-prep chromatography column (Bio-Rad Inc.). After the flow through fraction was collected, the column was washed with 10 vol. Buffer E (50 mM Tris.HCl, pH 7.5, 250 mM NaCl, 10% glycerol) containing 25 mM imidazole, and eluted successively with buffer E containing 100 mM imidazole (3 × 1.5 vol.) and Buffer E containing 300 mM imidazole (3 × 1.5 vol.). All wash and elution fractions were collected by gravity. Protein peaks were detected by sodium dodecyl sulphate-polyacrylamide gel electrophoresis (SDS-PAGE) and pooled for DNA-binding assays or further purification.

For FLAG affinity purification, extracts or Ni-NTA fractions were mixed with M2-agarose beads (20 vol. extract to 1 vol. beads) in FLAG buffer (50 mM Tris.HCl, pH 7.5, 250 mM NaCl, 10% glycerol, 0.1% NP-40, 2.5 mM MgCl_2_, 1 mM DTT) in microcentrifuge tubes. Following incubation with constant mixing on a rotator at 4°C for 2 h, the beads were washed five times with 20 vol. FLAG(150) buffer (same as FLAG buffer except that it contains 150 mM NaCl), and then the bound proteins eluted with 2.5 vol. FLAG(150) containing 0.2 mg/ml FLAG_3_ peptide. For glutathione-affinity purification, extracts or Ni-NTA fractions were mixed with glutathione-Sepharose beads (10 vol. extract to 1 vol. beads) in FLAG buffer for 2 h. After the same incubation and washing procedure as that for FLAG-antibody beads, the glutathione-bound proteins were eluted with 2.5 vol. FLAG(150) buffer containing 15 mM reduced glutathione.

For glycerol gradient, 200 μl of the indicated protein complex (∼0.2 to 2 μM) purified by affinity chromatography was applied to a 5 ml glycerol gradient (50 mM Tris-HCl, pH 7.5, 150 mM NaCl, 2 mM DTT, 0.1% Triton X-100, 15–30% glycerol). The gradient was subjected to centrifugation in an AH-650 rotor (Sorvall) at 4°C and 42,000 r.p.m. for 20 h, and 27 fractions were collected and analyzed by SDS-PAGE and Western.

### Protein concentration determination and western blot analysis

The concentrations of purified Cdc13s were estimated by SDS-PAGE and Coomassie staining; defined levels of bovine serum albumin (BSA) were applied to the same gel and their staining intensities used to construct a standard curve for protein concentration determination.

Western analysis was performed according to the ProtoBlot® II alkaline phosphatase (AP) System (Promega Corp.). The nitrocellulose membranes carrying transferred proteins were first incubated with primary antibodies (anti-His_6_ [Santa Cruz Biotechnology, Inc.] at 1:1000 dilution; anti-GST [GE Healthcare, Inc.] at 1:1000 dilution or anti-FLAG [Sigma-Aldrich Co.] at 1:5000 dilution) for 1 h, followed by incubation with alkaline phosphatase-conjugated secondary antibodies (anti-rabbit IgG, anti-goat IgG or anti-mouse IgG [Sigma-Aldrich Co.] at 1:5000 dilution) for 30 min. The antibody-bound proteins were visualized in nitro blue tetrazolium (NBT) and 5-bromo-4-chloro-3-indolyl-phosphate (BCIP)-containing buffers according to the manufacturer's instructions (Promega Corp.).

### Gel electrophoretic mobility shift analysis

Binding reactions contained 50 mM Tris-HCl (pH 7.5), 2 mM MgCl_2_, 10 mM NaCl, 1 mM spermidine, 1 mM DTT, 200 ng/μl poly(dI-dC), 5% glycerol and specified concentrations of probe and Cdc13 complexes. Following incubation at 25°C for 60 min, the reaction mixtures were electrophoresed through a 5% nondenaturing polyacrylamide gel (acrylamide : bis = 44 : 1) to resolve the free probe from the DNA–protein complex. Binding activity was analyzed using a Typhoon PhosphorImager and the ImageQuant software (GE Healthcare).

### Site-specific crosslinking analysis

The *Cp*Cdc13AB heterodimer (10 nM) was incubated with P^32^-labeled oligonucleotides containing 5-Iodo-2’-deoxyuridine substitutions (13 nM) in binding buffer (50 mM Tris-HCl, pH 7.5, 2 mM MgCl_2_, 10 mM NaCl, 1 mM spermidine, 1 mM DTT, 200 ng/μl poly(dI-dC), 5% glycerol) at 22°C for 1 h. The reaction mixtures were then placed on ice and irradiated with UV (Model UVM-57, UVP Inc.) for 20 min. After the addition of an equal volume of 2X SDS loading buffer, the samples were boiled for 5 min and analyzed by SDS-PAGE.

## RESULTS

### *Candida parapsilosis* Cdc13A and Cdc13B can each bind to itself and form heterodimers

Previous analysis of *C. albicans* Cdc13A and Cdc13B indicates that each paralog can self-associate as well as form heterodimers. To test the generality of this conclusion, we examined the abilities of *C. parapsilosis* Cdc13A (systematic name at the *Candida* genome database: CPAR2_105700) and Cdc13B (CPAR2_602150) to form homo-oligomers and/or hetero-oligomers in co-expression/pull down experiments. Briefly, we prepared extracts from *E. coli* strains expressing combinations of His-SUMO tagged and GST-tagged Cdc13s, and then subjected the extracts to pull down assays using GST-Sepharose. This investigation revealed significant binding of His-SUMO tagged Cdc13A to GST-tagged Cdc13A, as well as binding between differently tagged Cdc13Bs (Supplementary Figure S1, lanes 1–4 and lanes 5–8, respectively). Likewise, co-expression of His-SUMO-Cdc13A and GST-Cdc13B-FLAG followed by Ni-NTA and FLAG affinity chromatography revealed the existence of AB complexes (Figure 1B). Interestingly, both *Cp*Cdc13A and *Cp*Cdc13B migrate as larger than expected proteins in SDS-PAGE (Supplementary Figure S2), which may be due to clusters of acidic residues in these proteins (see Table 1 for the calculated and experimentally determined sizes of proteins used in this study) (22). To examine the oligomeric state of these complexes, we first purified His-SUMO-Cdc13A and GST-Cdc13B-FLAG separately by Ni-NTA and glutathione affinity chromatography, respectively, and subjected each preparation to glycerol gradient analysis. Notably, each protein was found broadly throughout the gradient (from >500 kDa to ∼60 kDa), suggesting the formation of heterogeneous aggregates (data not shown). Next, we isolated the Cdc13AB complex from cells co-expressing both paralogs by sequential Ni-NTA and FLAG affinity chromatography. Centrifugation of this material over a glycerol gradient revealed co-migration of a portion of Cdc13A and Cdc13B at ∼150 kDa, suggesting the formation of heterodimers (Figure 1C). In addition, a peak that is consistent with monomeric Cdc13A was also detected. In support of our interpretation, the same level of each protein was found in the dimer fractions as indicated by SDS-PAGE and Coomassie staining analysis (Figure 1C). These results for *C. parapsilosis* Cdc13A and Cdc13B are similar to what has been described for the *C. albicans* homologs (20) and suggest that the AB dimer is generally more stable.

### High-affinity and sequence-specific recognition of *C. parapsilosis* telomere G-strand by the *Cp*Cdc13AB heterodimer

**Figure 2.**
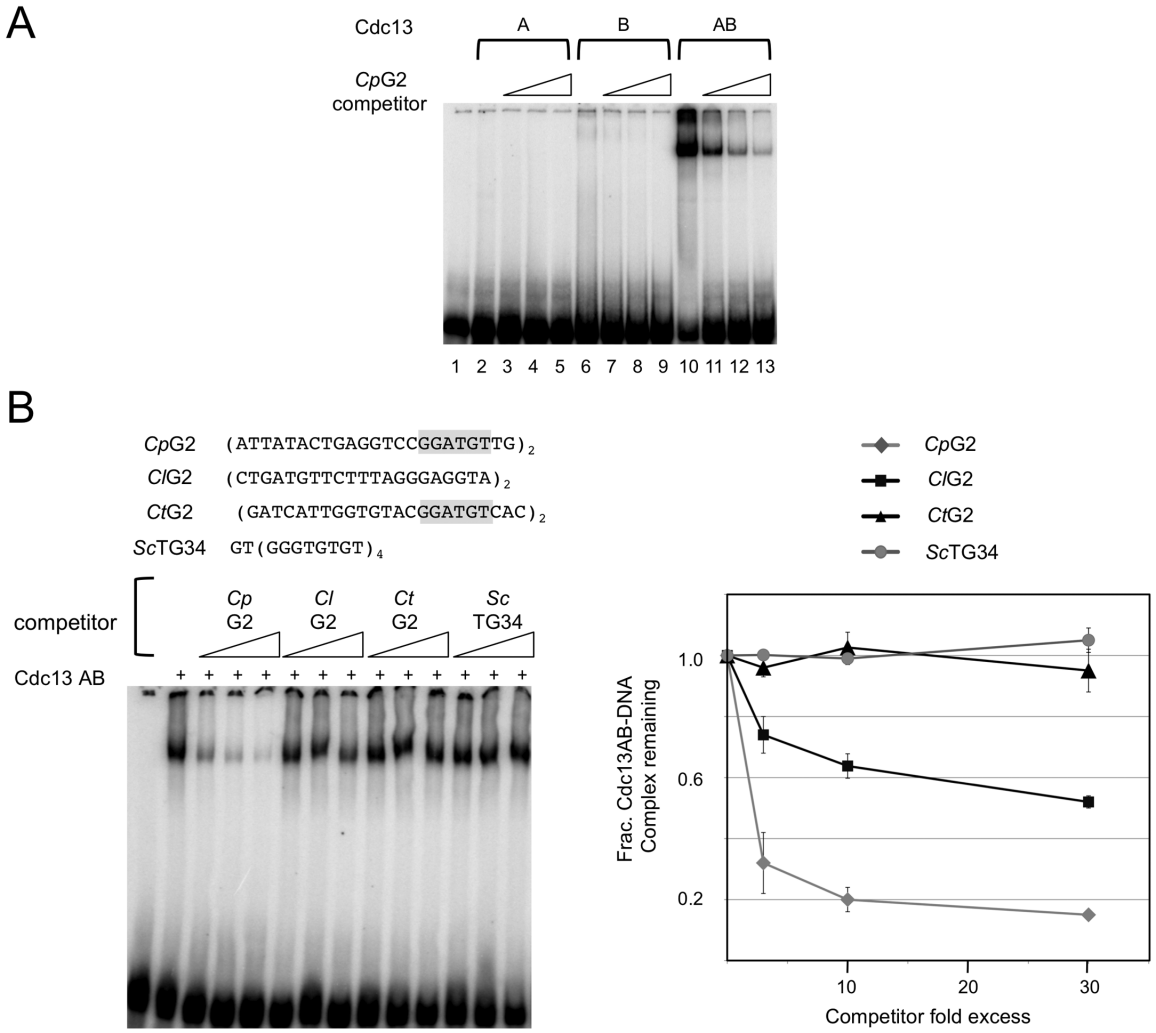
The DNA-binding activity of the *Cp*Cdc13AB complex. (**A**) *Cp*Cdc13A, Cdc13B and the Cdc13AB complex (∼30 nM) purified by affinity chromatography and glycerol gradients were tested for binding to the *Cp*G2 probe (7.5 nM). Three different concentrations of unlabeled *Cp*G2 oligos (at 3×, 9× and 30× the probe concentration) were added to the assays in order to judge the specificity of the interaction. (**B**) Left: Gel mobility shift assays were performed using the *Cp*G2 probe (7.5 nM) and the AB complex (10 nM). Increasing concentrations of unlabeled telomeric oligos (at 23, 75 and 225 nM) were added to the assays as competitors. *Cp, Cl, Ct* and *Sc* are abbreviations for *C. parapsilosis, C. lusitaniae, C. tropicalis* and *S. cerevisiae*. Right: The levels of the complex in the assays were normalized against that in the absence of competitor and plotted against the competitor/probe ratios.

Next we assayed the DNA-binding activity of the *Cp*Cdc13 AB dimer (purified on the glycerol gradient) using a probe that consists of two copies of the *Cp* telomere G-strand repeat (*Cp*G2, Figure 2A). For comparative purposes, the equivalent glycerol gradient fractions derived from processing of separately purified Cdc13A and Cdc13B proteins were also tested. Only the AB dimer fraction exhibited a robust DNA-binding activity, and the complex was efficiently competed by unlabeled *Cp*G2 oligo (Figure 2A, lanes 10–13). Titration analysis revealed an apparent *K*_d_ of ∼5–10 nM, suggesting a slightly higher affinity than previously reported for other *Candida* Cdc13-telomere DNA interaction (13,20,23). Removing both the His-SUMO and the GST tag by protease treatment increased the mobility of the DNA–protein complex, but did not alter the DNA-binding affinity of the AB dimer (Supplementary Figure S2). In addition, none of the glycerol gradient fraction exhibited significant C-strand binding activity, indicating that Cdc13s probably cannot interact with this complementary telomere strand (Supplementary Figure S3 and data not shown). We conclude that the Cdc13AB dimer binds selectively to the *C. parapsilosis* G-strand with high affinity. While we could not detect any DNA-binding activity in fractions that contain only Cdc13A or only Cdc13B, the aggregation propensity of each protein alone makes it difficult to draw clear conclusions concerning their biochemical activities.

To assess the sequence-specificity *Cp*Cdc13AB–DNA interaction, we tested the ability of three different heterologous telomere G-strands (*Cl*G2 (from *Candida lusitaniae*), *Ct*G2 (from *C. tropicalis*) and *Sc*TG34 (from *Saccharomyces cerevisiae*)) to compete for complex formation (Figure 2B). Interestingly, none of the heterologous G-strands competed efficiently, even though *Ct*G2 contains two copies of the consensus GGATGT sequence element previously shown to be the main determinant of Cdc13A–DNA interaction (Figure 2B). Approximately 20-fold higher concentration of *Cl*G2 was required to achieve 50% inhibition of complex formation, whereas *Ct*G2 and *Sc*TG34 were essentially unable to compete against *Cp*G2. These results differ substantially from those for *C. tropicalis* Cdc13AA, which binds strongly to both the *Ct* and *Cp* G-strands (12). Hence *Cp*Cdc13AB appears to bind telomere DNA in a more species-specific manner, possibly by recognizing sequence elements beyond the 6-nt consensus. We also tested the heterologous telomere oligos as probes in the binding assays and found that *Cp*Cdc13AB displayed less species-specificity in such assays (Supplementary Figure S4). While the reason for this is unclear, others have shown previously that OB fold DNA-binding domains are capable of adopting alternative conformations to bind different target sequences (24). Thus it is possible that in the absence of *Cp* telomere substrates, the AB dimer may bind heterologous repeats using alternative conformations.

**Figure 3.**
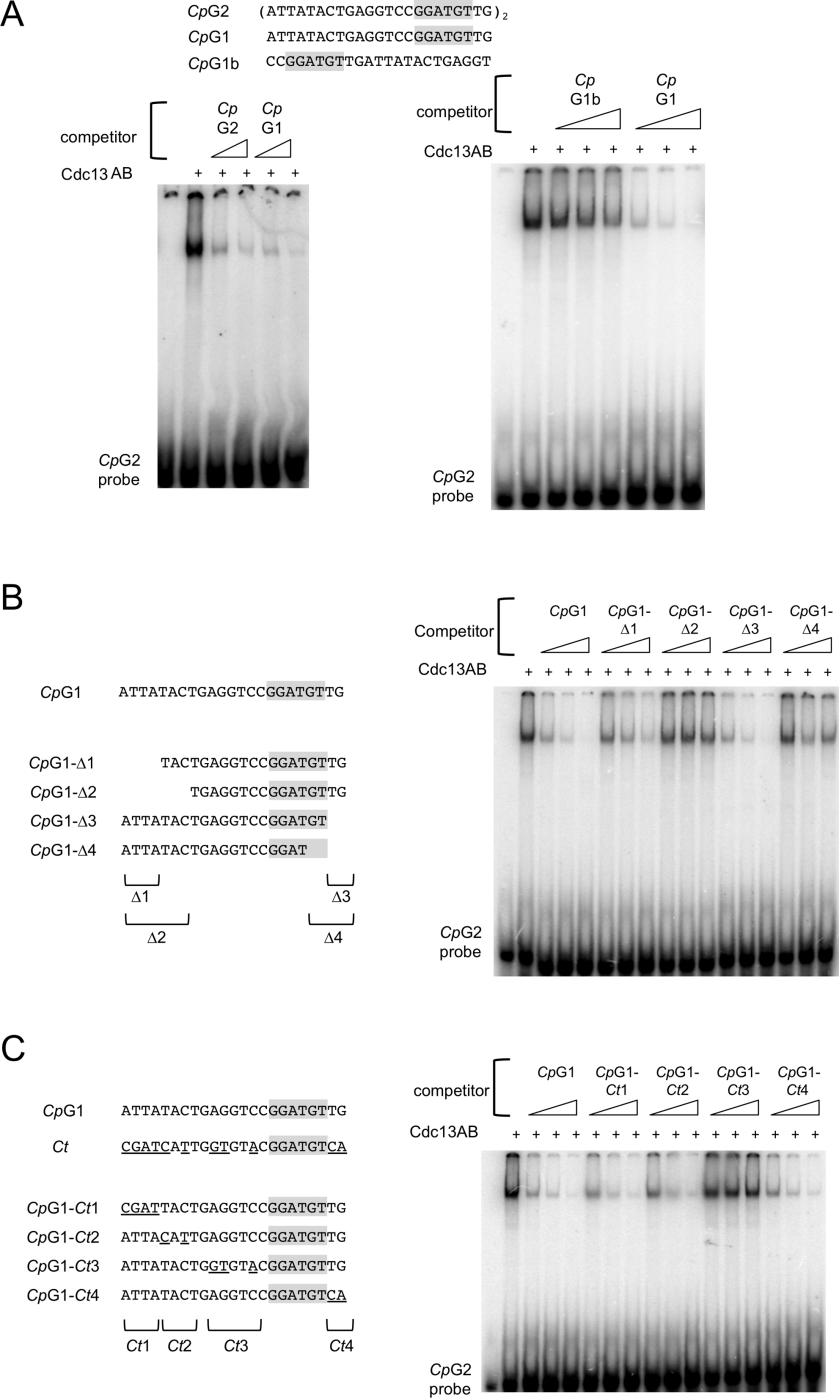
The sequence recognition property of the *Cp*Cdc13AB complex. (**A**) Left: Interaction between the *Cp*G2 probe (7.5 nM) and the *Cp*Cdc13AB complex (10 nM) was assessed in gel mobility shift assays. Unlabeled *Cp*G2 and *Cp*G1 oligos (at 250 and 750 nM) were added to the assays as competitors. Right: Interaction between the *Cp*G2 probe (7.5 nM) and the *Cp*Cdc13AB complex (10 nM) was assessed in gel mobility shift assays. Unlabeled *Cp*G1b and *Cp*G1 (at 23, 75 and 225 nM) were added to the assays as competitors. (**B**) Left: The truncation oligos used as competitors in the DNA-binding assays are illustrated. Right: Interaction between the *Cp*G2 probe (7.5 nM) and the *Cp*Cdc13AB complex (10 nM) was assessed in gel mobility shift assays. Unlabeled *Cp*G1-Δ1, Δ2, Δ3 and Δ4 oligos (at 23, 75 and 225 nM) were added to the assays as competitors. (**C**) Left: The hybrid *Cp/Ct* telomere oligos used as competitors in the DNA-binding assays are illustrated. Right: Interaction between the *Cp*G2 probe (7.5 nM) and the *Cp*Cdc13AB complex (10 nM) was assessed in gel mobility shift assays. Unlabeled *Cp*G1-*Ct*1, *Ct*2, *Ct*3 and *Ct*4 oligos (at 23, 75 and 225 nM) were added to the assays as competitors.

To probe the mechanism of recognition further, we examined two different permutations of the *C. parapsilosis* G-strand repeat unit to act as competitors (Figure 3A). In these two competitors, *Cp*G1 and *Cp*G1b, the 6-nt consensus is positioned in the 3′ and 5′ region of the oligonucleotides, respectively. Interestingly, *Cp*G1 competed as effectively as the *Cp*G2 oligo used as the standard probe, whereas *Cp*G1b was completely ineffective. This finding suggests that the AB complex may recognize, in addition to the consensus, nucleotides on the 5′ side of the consensus. To test this hypothesis, we eliminated different numbers of nucleotides from the 5′ and 3′ end of *Cp*G1, and tested the resulting oligos as competitors. In support of the need for the consensus element, deleting 4 nt from the 3′ side (i.e. removing 2 nt from the consensus) rendered the resulting oligo inactive as a competitor in the binding assays (Figure 3B, see results for *Cp*G1-Δ4). Interestingly, while removing 4 nt from the 5′ end (*Cp*G1-Δ1) had no effect, removing 7 nt (*Cp*G1-Δ2) abolished the ability of the resulting oligo to act as an effective competitor, suggesting that the AB complex may contact as many as 11 nt on the 5′ side of the consensus element.

Next, we investigated the molecular basis of the species-specific recognition of telomere G-strand by *Cp*Cdc13AB. Given the lower affinity of the *Cp*Cdc13AB dimer for the *C. tropicalis* G-strand, the dimer must recognize specifically some positions in the *Cp* repeat that are replaced by other nucleotides in the *Ct* repeat. Accordingly, we mutated clusters of nucleotides in *Cp*G1 by the corresponding nucleotides in the *Ct* repeat and tested the resulting oligos in competition assays (Figure 3C). Consistent with the results of the deletion analysis, replacing the four 5′-most nucleotides (*Cp*G1-*Ct*1) or the two 3′-most nucleotides (*Cp*G1-*Ct*4) did not affect the affinity of the oligo for the AB complex. In contrast, replacing 3 nt in the region immediately 5′ to the consensus element (*Cp*G1-*Ct*3) substantially reduced its affinity, suggesting that recognition at these three positions contributes to the ability of *Cp*Cdc13AB to discriminate between the cognate repeat and the *Ct* repeat. Interestingly, even though deleting nucleotides 5–7 of *Cp*G1 drastically impaired its binding to the AB dimer (see the results for *Cp*G1-Δ2 in Figure 3B), replacing these nucleotides by the corresponding nucleotides in the *Ct* repeat (*Cp*G1-*Ct*2) had no effect, suggesting that these positions are not bound by the AB dimer with strict sequence-specificity (Figure 3C).

To assess further the ability of *Cp*Cdc13AB to discriminate against heterologous G-strand, we examined the activities of the *C. orthopsilosis* (*Co*) and *C. metapsilosis* (*Cm*) G-strands in competition assays (Supplementary Figure S5). Like the *Ct* G-strand, the *Co* and *Cm* G-strands share the 6-nt consensus element, but differ in sequence from the *Cp* G-strand at several positions 5′ to the consensus (Supplementary Figure S5). Notably, both *Co*G1 and *Cm*G1 exhibit much weaker binding to the AB dimer than *Cp*G1 in competition assays. Together with the finding on the *Cp*G1-*Ct*3 oligo, the results suggest that at least 4 nt in the region 5′ to the consensus element are bound by *Cp*Cdc13AB in a sequence-specific manner, thus accounting for the ability of the heterodimer to discriminate against heterologous G-strand.

### Combinatorial recognition of the telomere G-strand by the *Cp*Cdc13AB dimer

**Figure 4.**
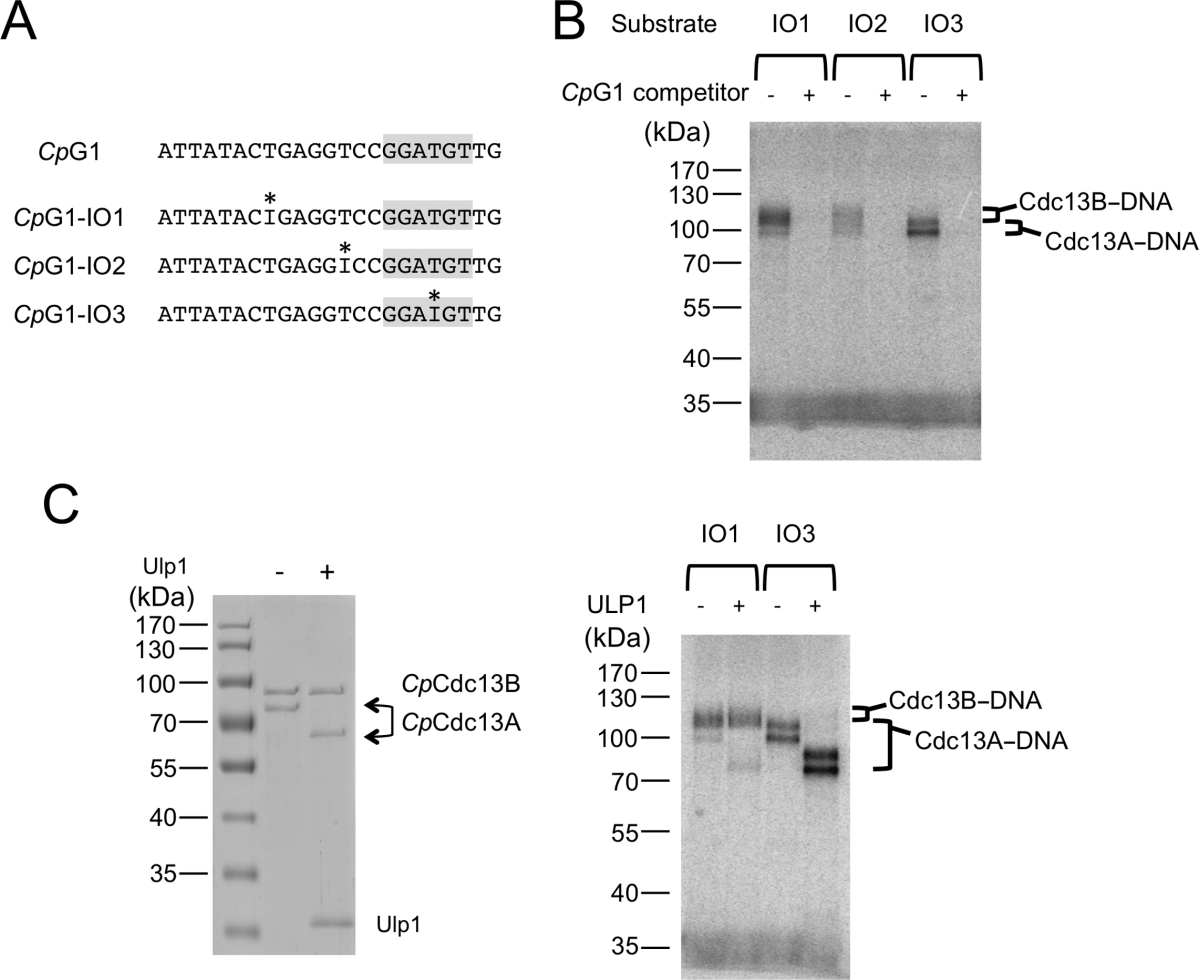
Combinatorial recognition of telomere G-strand by the *Cp*Cdc13AB dimer. (**A**) The oligonucleotides used for the site-specific crosslinking analysis are illustrated. The positions of the 5-Iodo-2’-deoxyuridine analog are indicated by asterisks. (**B**) The *Cp*Cdc13AB dimer was incubated with the indicated oligonucleotides (pre-labeled with P^32^) and subjected to UV irradiation. The covalent protein–DNA conjugates were separated from free DNA by SDS-PAGE and detected by PhosphorImager analysis. The specificity of the crosslinking reaction was tested by adding 100-fold excess of unlabeled *Cp*G1 competitor to the reactions. (**C**) Left: The *Cp*Cdc13AB dimer was subjected to Ulp1 treatment to eliminate the SUMO tag from the Cdc13A fusion protein. Right: Untreated or Ulp1-treated *Cp*Cdc13AB dimer was subjected to crosslinking assays using the indicated substrates.

Taking into consideration the previous finding that *C. tropicalis* Cdc13A recognizes the 6-nt consensus element, we surmise that Cdc13B may bind the region 5′ to the consensus. To gain direct physical evidence for the proposed combinatorial recognition mechanism, we subjected the *Cp*Cdc13AB–DNA complex to site-specific crosslinking analysis. Three thymidine residues in *Cp*G1 (at positions 8, 13 and 19) were individually replaced with 5-Iodo-2’-deoxyuridine, a photo-activatible analog, to yield the IO-1, IO-2 and IO-3 oligos (Figure 4A). The oligos were labeled with P^32^, incubated with the AB heterodimer and irradiated with long wave UV to generate covalent adducts. All three photoactive oligos cross-linked to proteins as judged by SDS-PAGE and PhosphorImager analysis, with the IO-1 and IO-3 generating higher levels of products than IO-2 (Figure 4B). The products produced by IO-3 are slightly smaller than those by IO-1 and IO-2, suggesting that the former may preferentially cross-link to the smaller *Cp*Cdc13A fusion protein. Consistent with this hypothesis, all the adducts generated by IO-3 were reduced in size upon prior Ulp1 treatment, which removes the SUMO tag from the *Cp*Cdc13A fusion protein (Figure 4C). In contrast, the majority of products yielded by IO-1 were unaffected by Ulp1 treatment, suggesting that they represent *Cp*Cdc13B-DNA adducts. These results are entirely compatible with the notion that Cdc13A and Cdc13B make physical contacts to the 3′ and 5′ region of the *Cp* telomere repeat, respectively.

An interesting issue raised by the proposed ‘combinatorial’ mechanism of recognition is whether the AB complex can tolerate nucleotide insertions between the target sites for the two protein subunits. To address this issue, we interpolated 1, 6 and 12 nt between the putative A and B site in *Cp*G1, and tested the resulting oligos in competition assays (Supplementary Figure S6). Notably, inserting just 1 nt reduced the affinity of the oligo by ∼5–10-fold, while inserting 6 or 12 nt rendered the resulting oligos essentially inactive in the competition assays. Thus, the DNA-binding surfaces of the Cdc13A and B subunits appear to be tightly juxtaposed to each other, and are evidently unable to accommodate changes in the distance between the target sites.

### Association between the OB4s of *Cp*Cdc13A and CpCdc13B, as well as that between the DBDs

**Figure 5.**
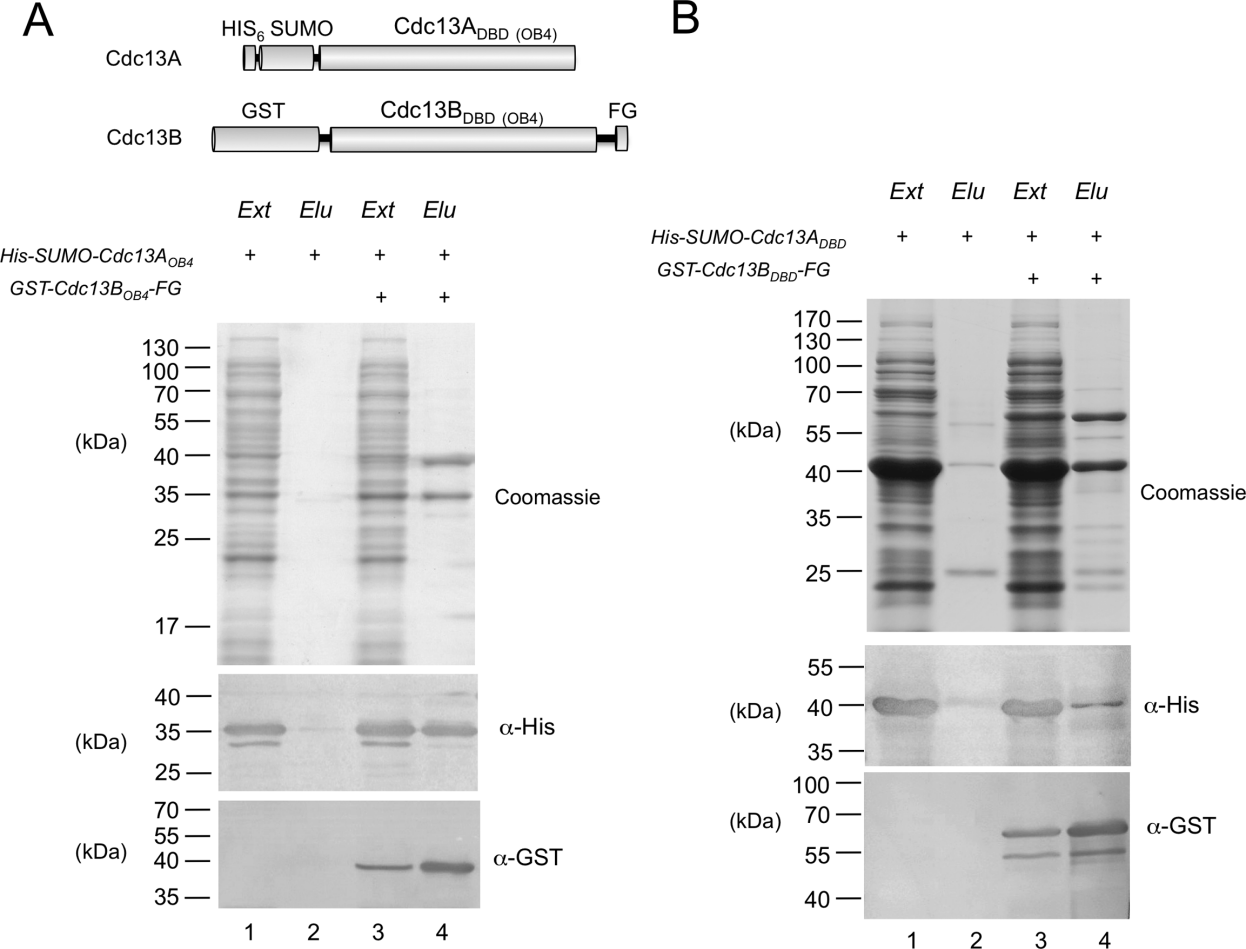
Complex formation between the OB4s and DBDs of *Cp*Cdc13A and CpCdc13B. (**A**) Top: The fusion proteins used in the co-expression and anti-FLAG affinity purification analyses are illustrated. Bottom: Fusions proteins containing the OB4 domains were expressed alone or co-expressed in *E. coli*, and the extracts were subjected to FLAG affinity chromatography. The protein contents of the cell extracts (Ext) and FLAG elution fractions (Elu) were analyzed by SDS-PAGE and Coomassie staining. The levels of the His_6_-SUMO fusion protein (the target protein) and the GST-FLAG fusion protein (the bait protein) in the indicated cell extracts (Ext) and elution fractions (Elu) were also analyzed by western using anti-His_6_ and anti-GST antibodies. (**B**) Fusion proteins containing the DBD domains were expressed alone or co-expressed in *E. coli*, and the extracts were subjected to FLAG affinity chromatography. The protein contents of the cell extracts (Ext) and FLAG elution fractions (Elu) were analyzed by SDS-PAGE and Coomassie staining. The levels of the His_6_-SUMO fusion protein (the target protein) and the GST-FLAG fusion protein (the bait protein) in the indicated cell extracts (Ext) and elution fractions (Elu) were also analyzed by western using anti-His_6_ and anti-GST antibodies.

Having shown heterodimerization of the Cdc13A and Cdc13B paralogs and characterized the DNA-binding activity of the heterodimer, we next sought to determine the contributions of the DBD and OB4 domains of each protein to these properties. First, we used co-expression of tagged domains and affinity purification to assess the ability of these domains to form complexes. Remarkably, both the DBD and OB4 domain of Cdc13A can form a complex with the corresponding domain of Cdc13B in this analysis, suggesting that the full-length heterodimer contains two sets of inter-domain interactions (Figure 5A and B).

To analyze in more detail the stoichiometry of the DBD and OB4 complexes, we subjected the affinity-purified complexes to glycerol gradient analyses. Interestingly, the AB_OB4_ complex sedimented as a single peak with an estimated size of ∼90 kDa, which is close to that predicted for a heterodimer (Supplementary Figure S7A). In contrast, the two DBDs in the affinity-purified fraction both sedimented broadly in the glycerol gradient, consistent with propensity to aggregate and to form heterogeneous complexes. Notably, a peak corresponding to monomeric B_DBD_ can be detected, suggesting that the complexes may also be unstable and prone to dissociation (Supplementary Figure S7A). To assess the stability of these complexes further, we subjected the gradient fractions corresponding to the AB_DBD_ and AB_OB4_ dimers to dilution and a second round of glycerol gradient analysis (Supplementary Figure S7B). Over the second gradient, the AB_OB4_ complex again behaved as a homogeneous heterodimer. In contrast, while the majority of both A_DBD_ and B_DBD_ co-sedimented as heterodimers (Supplementary Figure S7B, marked by a thick arrow), a portion of each sedimented as a monomer (marked by thin arrows), indicating partial dissociation of the complex. We surmise that the interactions between the OB4 domains may play a more important role than the interactions between the DBDs in maintaining the stability of the full-length heterodimer.

### DNA binding by the AB_DBD_ complex

**Figure 6.**
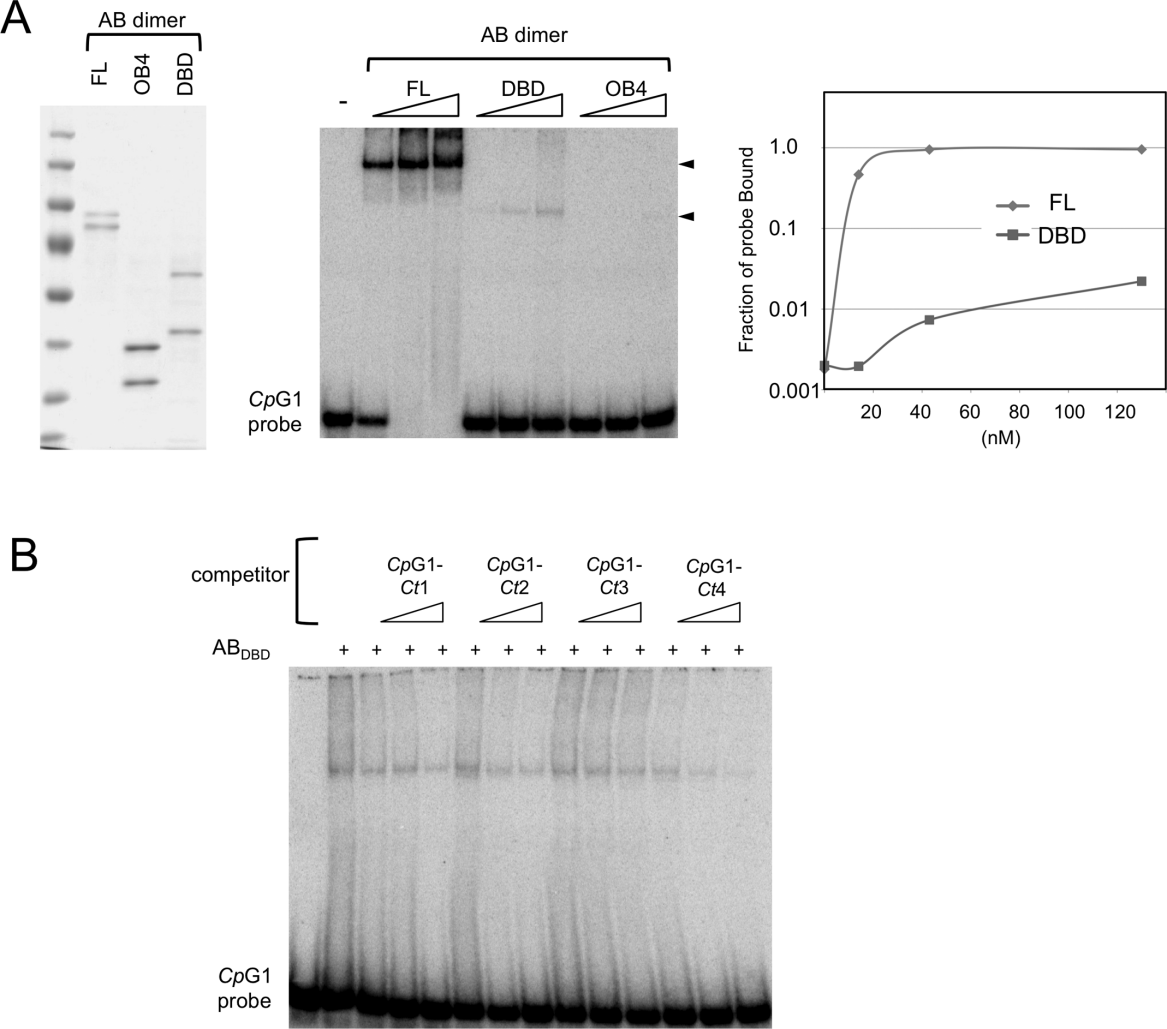
Weak but sequence-specific DNA-binding by the AB_DBD_ complex. (**A**) Left: The glycerol gradient fractions used for the DNA-binding assays were analyzed by SDS-PAGE and Coomassie staining. Middle: The interaction between the *Cp*G1 probe (7.5 nM) and the *Cp*Cdc13AB, AB_DBD_ and AB_OB4_ complexes (13, 40 and 120 nM) was assessed in gel mobility shift assays. Complexes of different mobility were generated by *Cp*Cdc13AB and AB_DBD_ (arrowheads). Right: The results of the binding assays were quantified and plotted. (**B**) The interaction between the *Cp*G1 probe (7.5 nM) and the AB_DBD_ complex (60 nM) was assessed in gel mobility shift assays. Unlabeled *Cp*G1-*Ct*1, *Ct*2, *Ct*3 and *Ct*4 oligos (at 23, 75 and 225 nM) were added to the assays as competitors.

To determine the contributions of the DBD and OB4 domains to the DNA-binding activity of the full-length complex, we first assayed the glycerol gradient fractions obtained from the AB_DBD_ and AB_OB4_ complexes for G-strand binding activities. Consistent with all previous studies, no activity was detected throughout the OB4 gradient. In contrast, a weak DNA-binding activity was detected in the DBD gradient in the ∼100 kDa range (similar to that for the AB_DBD_ dimer). As expected, the AB_DBD_–DNA complex has a mobility that is different from the AB–DNA complex (Figure 6A, the two complexes marked by arrowheads). Side-by-side comparison of the binding activity of the full length and DBD complexes indicates that the former has a *K*_d_ for DNA (∼10 nM) that is at least 100-fold lower than the latter has (Figure 6A). Despite the dramatic difference in binding affinity, the AB_DBD_ complex exhibits similar binding specificity as the full-length heterodimer. In particular, in competition assays, the *Cp*G1-*Ct*3 oligo was less active in binding AB_DBD_ than the other hybrid oligos, just like the results for the full-length AB complex (Figure 6B). Taken together, our data support the notion that the G-strand recognition specificity of *Cp*Cdc13AB is inherent to the DBD dimer, and that the OB4 dimer contributes to binding affinity by stabilizing the complex consisting of full-length proteins.

## DISCUSSION

Previous analysis of Cdc13s in *C. albicans* and *C. tropicalis* suggests that this family of proteins may recognize primarily conserved sequence elements in diverse *Candida* telomere repeats, and may not have evolved strong preferences for the cognate telomere G-strands. This notion was in agreement with conclusions derived from comparative analysis of *S. cerevisiae* and *Saccharomyces castellii* Cdc13s, which likewise highlight the importance of conserved nucleotides in high-affinity binding (10,25). However, the current study indicates that at least for one *Candida* species, the Cdc13 proteins have achieved highly tailored, species-specific binding to the cognate telomere repeat unit by forming heterodimers and by utilizing a combinatorial mechanism of recognition. The implications of these findings are discussed.

### The dimerization of Cdc13s

Dimerization is both a conserved and a malleable property of Cdc13 homologues; all Cdc13s appear capable of forming either homodimers or heterodimers (or both), but the domains responsible for dimerization and the functions of dimerization differ among Cdc13s. For example, the OB1 of *Sc*Cdc13 but not *Cg*Cdc13 has a strong propensity to dimerize, and dimerization of *Sc*Cdc13 OB1 results in a binding site for Pol1. Dimerization of *Ct*Cdc13A through its OB4 domain, by contrast, is required for high-affinity binding of this homologue to the cognate G-strand (12). Interestingly, each OB fold domain of Cdc13, with the exception of DBD, was previously shown to form dimers in at least one homologue. Indeed, crystal structures of dimeric OB folds are available for the OB1 and OB2 of *Sc*Cdc13, as well as for the OB4 of *Cg*Cdc13 (12,17,18). We now demonstrate that even some DBD domains may be capable of dimerization (albeit relatively weak dimerization), thus raising the possibility that all full-length Cdc13s may be in the form of a series of dimers.

**Figure 7.**
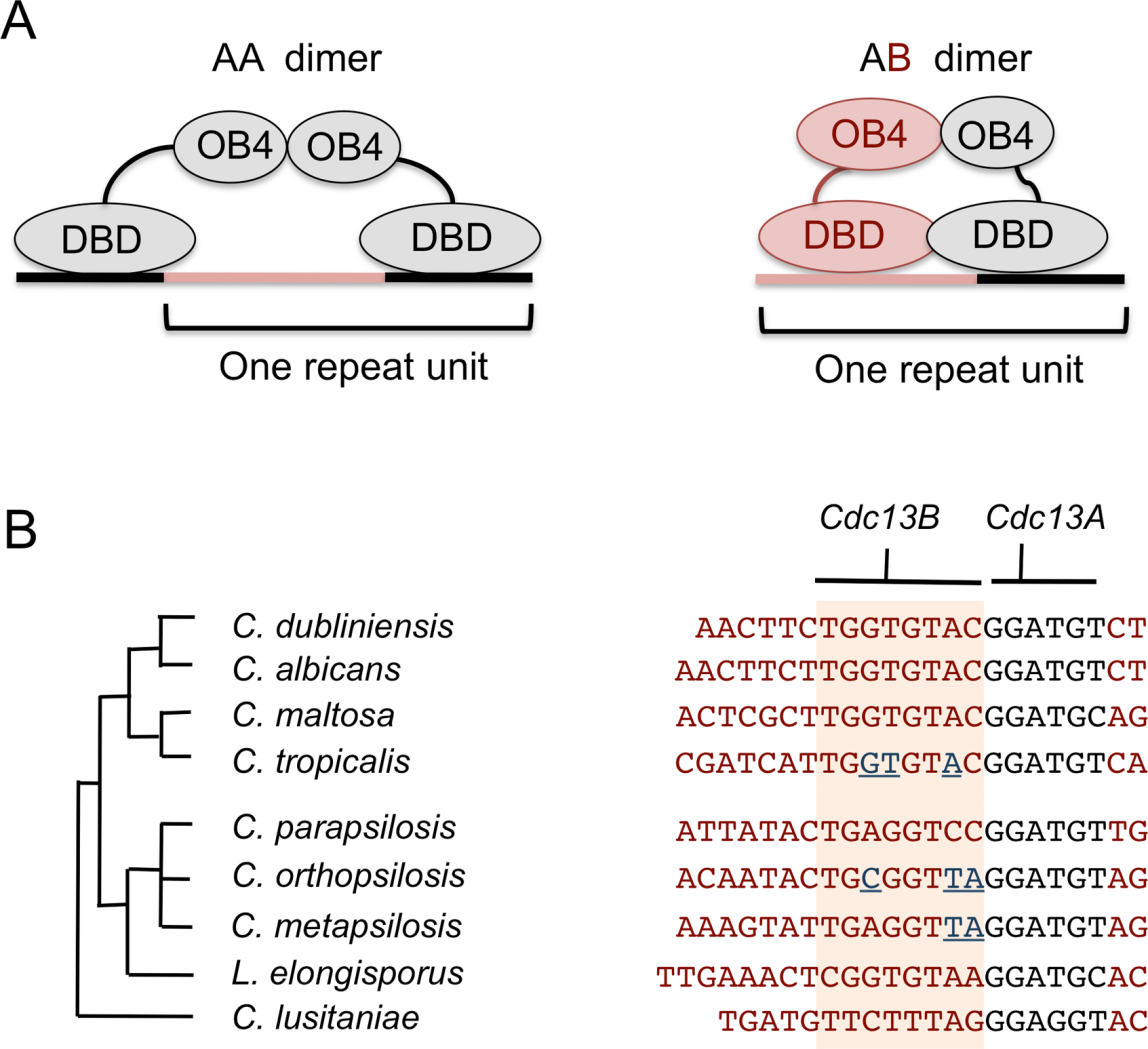
Models for Cdc13-telomere interaction. (**A**) The proposed models for the *Ct*Cdc13AA–DNA and *Cp*Cdc13AB–DNA complexes are schematically illustrated. (**B**) Left: The phylogenetic relationship among the *Candida* species is displayed. Right: The telomere repeat units in these organisms are shown along with the proposed Cdc13A and Cdc13B target sites. The region bound by Cdc13B is shaded in orange, and the nucleotides in *C. tropicalis, C. orthopsilosis* and *C. metapsilosis* G-strand that cause reduced binding by *Cp*Cdc13AB are shown in blue and underlined.

The high-affinity binding of the *C. parapsilosis* AB heterodimer and the lack of detectable DNA-binding by the putative AA and BB complexes suggest that the heterodimer is the physiologically relevant form of Cdc13 in this organism. Whether this is true of other *Candida* species is not yet clear. Both the *C. albicans* and *C. tropicalis* Cdc13AA complexes bind with moderate affinity to the cognate telomere repeats, as long as two copies of the 6-nt consensus are present in the substrate. Conceivably the AA dimers could localize to telomeres if long G-tails are present and if the dimers are sufficiently abundant (Figure 7A). Direct analysis of Cdc13 complexes in *Candida* cells and extracts will be necessary to confirm the existence and relevance of alternative complexes.

### Sequence-specific recognition of *C. parapsilosis* G-tail by the CpCdc13AB heterodimer

Our observations indicate that the high affinity and sequence specificity of the *Cp*Cdc13AB–telomere interaction is achieved through a combinatorial mechanism in which the Cdc13A_DBD_–DNA and Cdc13B_DBD_–DNA interactions work cooperatively to generate a stable complex (Figure 7). In particular, truncating either the A subunit or the B subunit target site drastically reduced binding affinity (Figure 3). Moreover, mutating just 3 nt in the B subunit target site substantially reduced binding. In the high-affinity complex, the DNA-binding surfaces of the A and B subunits appear to be tightly juxtaposed to each other; inserting just 1 nt in between the target sites substantially reduced binding, whereas inserting 6 or more nucleotides abolished binding (Supplementary Figure S6). The DNA-binding surfaces of the two subunits also have a defined spatial orientation; the B target site must be positioned 5′ to the A target site in order to support high-affinity binding (Figure 3A). Our failure to detect any DNA-binding by the *C. parapsilosis* A or B subunit alone (despite their ability to form homo-oligomers) suggests that the intrinsic affinity of either DBD for DNA in isolation may be extremely low, and that the protein–protein interaction between the DBDs in the heterodimer may contribute to DNA-binding (e.g. by triggering some conformational change in the DNA-binding surface). Thus, the highly cooperative nature of the *Cp*Cdc13AB–DNA complex appears to be quite distinct from that of the *Ct*Cdc13AA–DNA complex, where the two Cdc13A_DBD_–DNA interactions appear to be more independent of each other and can be separated by variable numbers of nucleotides (Figure 7) (12). Continued analyses of these complexes and additional examples of *Candida* Cdc13–DNA interactions should lead to a broader understanding of strategies for ssDNA recognition.

### Co-evolution of Cdc13s and telomere repeat sequence in Candida: the capacity of Cdc13 to evolve new sequence-specificity

Despite the extraordinary variability of the telomere repeat units in Sacchromycotina yeast, it has long been recognized that these repeats share a conserved motif that may be the primary recognition target of proteins that binds to telomeres (6). Detailed investigation of the binding properties of *S. cerevisiae* and *S. castellii* Cdc13 and Rap1 are mostly consistent with this notion, i.e. the conserved nucleotides appear to be the most important ones for high-affinity binding to proteins (8–10). A possible implication for the ‘conserved’ recognition mechanism is that the DNA-binding domains of Rap1 and Cdc13 may have very limited capacity to acquire new sequence-specificity through mutational changes (at least during the time window after the divergence of the *Saccharomyces* and *Candida* lineages, which is estimated to be ∼300 million years (26,27)). However, our observations indicate that in the case of the DBD of *C. parapsilosis* Cdc13B, highly species-specific sequence recognition has been achieved. This species-specificity is evidently based on selective interactions with 3 nt in the target site that are unique to the *C. parapsilosis* repeat and that are absent from most other repeats (Figure 7). Thus, the ability of the Cdc13 DBDs to evolve new recognition specificity is probably greater than previously realized. Whether other *Candida* Cdc13s are also able to discriminate against non-cognate telomere sequences is an interesting issue for future investigations. A broad survey of the DNA-binding specificity of multiple *Candida* Cdc13s should provide insights into the ‘evolvability’ of this DNA-binding fold, and offer new lessons on the mechanisms of ssDNA recognition. In this regard, we note that even though this study highlights the potential of Cdc13B to acquire unique sequence specificity, the potential of Cdc13A to do so should not be discounted. Even though the GGATGT element is shared exactly by many *Candida* species, a few organisms possess slight variants (i.e. GGATGC in *Candida maltosa* and *Lodderomyces elongisporus* and GGAGGT in *C. lusitaniae*) (Figure 7B). Assessing the DNA-binding specificity of Cdc13A in these particular organisms should be informative.

### The roles of gene duplication and protein multimerization in telomere evolution

As noted in a previous work, the duplication of G-tail binding proteins has occurred multiple times in different phyla, suggesting that it can confer substantial evolutionary advantages (20). One possible advantage, as suggested by the current observations, is increased capacity to adapt to alterations in telomere repeat sequence. By forming heterodimers and hence an extended DNA-binding surface, the Cdc13 complex may become less reliant on recognition of individual nucleotides for high-affinity binding. Thus, when the telomere repeats acquire mutations, the Cdc13 complex may retain sufficient affinity for the mutated sequence to allow survival, and during the subsequent course in evolution, acquire enough compensatory changes to optimize binding affinity. Perhaps the existence of Cdc13 dimers in the Saccharomycotina ancestor provided the enabling condition for the dramatic divergence of telomere repeats in its descendants.

A further speculation concerns the somewhat puzzling ability of at least some Cdc13A proteins to form homodimers that possess moderate affinity for telomeric DNA. Given the potential ability of the heterodimers to achieve greater sequence-specificity by recognizing two distinct target sites, it is unclear why the capacity for homodimerization has not been lost in evolution. While there are clearly alternative rationales, one advantage of preserving the homodimer is that it could serve a back-up function in the case of drastic telomere sequence changes; as long as the 6-nt consensus element is retained, the homodimer will be able to localize to telomeres and mediate its protective functions. In short, by maintaining a degree of flexibility in their dimerization properties, the Cdc13 paralogs were able to elaborate alternative complexes that allow the organism to cope with challenges posed by the rapidly evolving telomere repeats.

## SUPPLEMENTARY DATA

Supplementary Data are available at NAR Online.

SUPPLEMENTARY DATA

## References

[1] Palm W., de Lange T. (2008). How shelterin protects mammalian telomeres. Annu. Rev. Genet..

[2] O'Sullivan R.J., Karlseder J. (2010). Telomeres: protecting chromosomes against genome instability. Nat. Rev. Mol. Cell Biol..

[3] Jain D., Cooper J.P. (2011). Telomeric strategies: means to an end. Annu. Rev. Genet..

[4] Podlevsky J.D., Bley C.J., Omana R.V., Qi X., Chen J.J. (2008). The telomerase database. Nucleic Acids Res..

[5] Lue N.F. (2010). Plasticity of telomere maintenance mechanisms in yeast. Trends Biochem. Sci..

[6] McEachern M.J., Blackburn E.H. (1994). A conserved sequence motif within the exceptionally diverse telomeric sequences of budding yeasts. Proc. Natl Acad. Sci. U.S.A..

[7] Gunisova S., Elboher E., Nosek J., Gorkovoy V., Brown Y., Lucier J.F., Laterreur N., Wellinger R.J., Tzfati Y., Tomaska L. (2009). Identification and comparative analysis of telomerase RNAs from Candida species reveal conservation of functional elements. RNA.

[8] Wahlin J., Cohn M. (2000). Saccharomyces cerevisiae RAP1 binds to telomeric sequences with spatial flexibility. Nucleic Acids Res..

[9] Rhodin Edso J., Gustafsson C., Cohn M. (2011). Single- and double-stranded DNA binding proteins act in concert to conserve a telomeric DNA core sequence. Genome Integr..

[10] Rhodin Edso J., Tati R., Cohn M. (2008). Highly sequence-specific binding is retained within the DNA-binding domain of the Saccharomyces castellii Cdc13 telomere-binding protein. FEMS Yeast Res..

[11] Yu E.Y., Yen W.F., Steinberg-Neifach O., Lue N.F. (2010). Rap1 in Candida albicans: an unusual structural organization and a critical function in suppressing telomere recombination. Mol. Cell. Biol..

[12] Yu E.Y., Sun J., Lei M., Lue N.F. (2012). Analyses of Candida Cdc13 orthologues revealed a novel OB fold dimer arrangement, dimerization-assisted DNA binding, and substantial structural differences between Cdc13 and RPA70. Mol. Cell. Biol..

[13] Mandell E.K., Gelinas A.D., Wuttke D.S., Lundblad V. (2011). Sequence-specific binding to telomeric DNA is not a conserved property of the Cdc13 DNA binding domain. Biochemistry.

[14] Baumann P., Price C. (2010). Pot1 and telomere maintenance. FEBS Lett..

[15] Giraud-Panis M.J., Teixeira M.T., Geli V., Gilson E. (2010). CST meets shelterin to keep telomeres in check. Mol. Cell.

[16] Sun J., Yu E.Y., Yang Y., Confer L.A., Sun S.H., Wan K., Lue N.F., Lei M. (2009). Stn1-Ten1 is an Rpa2-Rpa3-like complex at telomeres. Genes Dev..

[17] Sun J., Yang Y., Wan K., Mao N., Yu T.Y., Lin Y.C., Dezwaan D.C., Freeman B.C., Lin J.J., Lue N.F. (2011). Structural bases of dimerization of yeast telomere protein Cdc13 and its interaction with the catalytic subunit of DNA polymerase alpha. Cell Res..

[18] Mason M., Wanat J.J., Harper S., Schultz D.C., Speicher D.W., Johnson F.B., Skordalakes E. (2012). Cdc13 OB2 dimerization required for productive Stn1 binding and efficient telomere maintenance. Structure.

[19] Mitton-Fry R., Anderson E., Hughes T., Lundblad V., Wuttke D. (2002). Conserved structure for single-stranded telomeric DNA recognition. Science.

[20] Lue N.F., Chan J. (2013). Duplication and functional specialization of the telomere-capping protein Cdc13 in Candida species. J. Biol. Chem..

[21] Mossessova E., Lima C.D. (2000). Ulp1-SUMO crystal structure and genetic analysis reveal conserved interactions and a regulatory element essential for cell growth in yeast. Mol. Cell.

[22] Shi Y., Mowery R.A., Ashley J., Hentz M., Ramirez A.J., Bilgicer B., Slunt-Brown H., Borchelt D.R., Shaw B.F. (2012). Abnormal SDS-PAGE migration of cytosolic proteins can identify domains and mechanisms that control surfactant binding. Protein Sci..

[23] Lue N.F., Zhou R., Chico L., Mao N., Steinberg-Neifach O., Ha T. (2013). The telomere capping complex CST has an unusual stoichiometry, makes multipartite interaction with G-tails, and unfolds higher-order G-tail structures. PLoS Genet..

[24] Theobald D.L., Schultz S.C. (2003). Nucleotide shuffling and ssDNA recognition in Oxytricha nova telomere end-binding protein complexes. EMBO J..

[25] Hughes T.R., Weilbaecher R.G., Walterscheid M., Lundblad V. (2000). Identification of the single-strand telomeric DNA binding domain of the Saccharomyces cerevisiae Cdc13 protein. Proc. Natl Acad. Sci. U.S.A..

[26] Hedges S.B. (2002). The origin and evolution of model organisms. Nat. Rev. Genet..

[27] Pesole G., Lotti M., Alberghina L., Saccone C. (1995). Evolutionary origin of nonuniversal CUGSer codon in some Candida species as inferred from a molecular phylogeny. Genetics.

