# Supplementary material for: Combinatorial recognition of a complex telomere repeat sequence by the Candida parapsilosis Cdc13AB heterodimer
Source: Nucleic Acids Res. 2015 Feb 8;43(4):2164–76. doi: 10.1093/nar/gkv092 (PMC4344524; doi:10.1093/nar/gkv092)
Supplement: SUPPLEMENTARY DATA [file supp_43_4_2164__index.html]

Combinatorial recognition of a complex telomere repeat sequence by the Candida parapsilosis Cdc13AB heterodimer — SUPPLEMENTARY DATA 

# Combinatorial recognition of a complex telomere repeat sequence by the *Candida parapsilosis* Cdc13AB heterodimer

## SUPPLEMENTARY DATA

**Files in this Data Supplement:**

- SUPPLEMENTARY DATA
