# Supplementary material for: Combinatorial recognition of a complex telomere repeat sequence by the Candida parapsilosis Cdc13AB heterodimer
Source: Nucleic Acids Res. 2015 Feb 8;43(4):2164–76. doi: 10.1093/nar/gkv092 (PMC4344524; doi:10.1093/nar/gkv092)
Supplement: SUPPLEMENTARY DATA [file supp_gkv092_Supp_data_all_V2.pdf]

Supplementary Data

Steinberg-Neifach et al.

Fig. S1

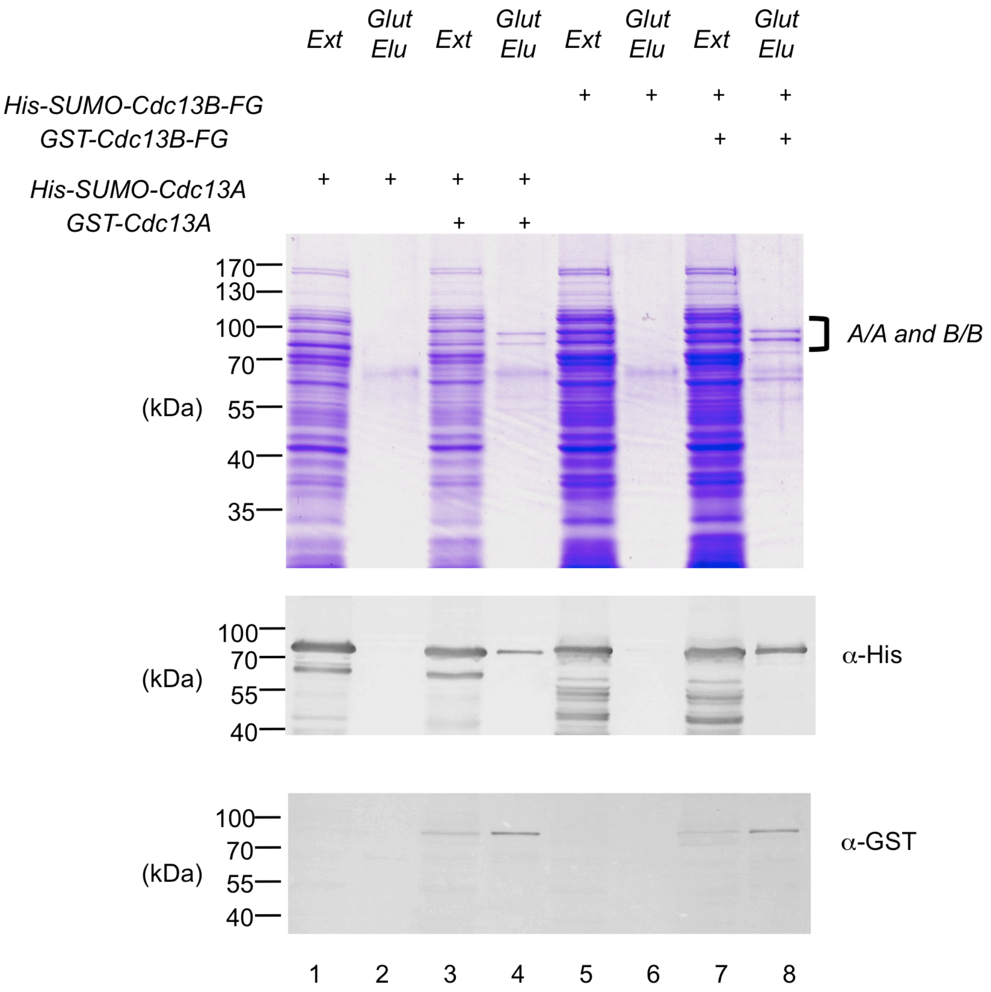

Fig. S1. Self-association by CpCdc13A and CpCdc13B

The indicated fusion proteins were expressed alone or co-expressed in *E. coli*, and the extracts were subjected to Glutathione-Sepharose affinity chromatography. The protein contents of the cell extracts (Ext) and Glutathione elution (Glut Elu) fractions were analyzed by SDS-PAGE and Coomassie staining. The levels of the His_6-SUMO fusion proteins (the target protein) and the GST-FLAG fusion proteins (the bait protein) in the indicated cell extracts and elution fractions were also analyzed by Western using anti-His_6 and anti-GST antibodies.

Fig. S2

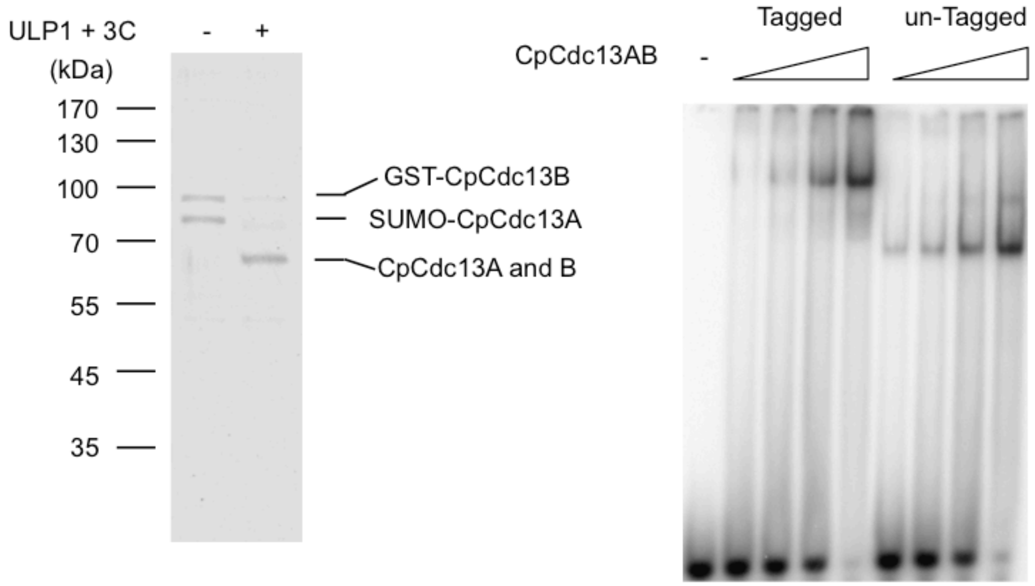

Fig. S2. Comparison of the G-strand binding activity of the CpCdc13AB dimer before and after tag removal

(Left) SDS-PAGE and Coomassie staining of the *Cp*Cdc13AB dimer before and after combined ULP1 and 3C treatment. Note that the untagged Cdc13A (52.7 kD) and Cdc13B (53.4 kD) migrate to the same position in the gel. Both proteins are quite acidic, with pIs round 5.0, which may explain their anomalously slow migration in the gel.

(Right) The CpG1 probe (7.5 nM) was incubated with increasing concentrations of tagged or untagged *Cp*Cdc13AB (5, 10, and 20 nM) and complex formation was monitored by native gel electrophoresis.

Fig. S3

*Cp*G1 ATTATACTGAGGTCCGGATGTTG

*Cp*C1 CAACATCCGGACCTCAGTATAAT

*Cp*C2 $(\text{CAACATCCGGACCTCAGTATAAT})_2$

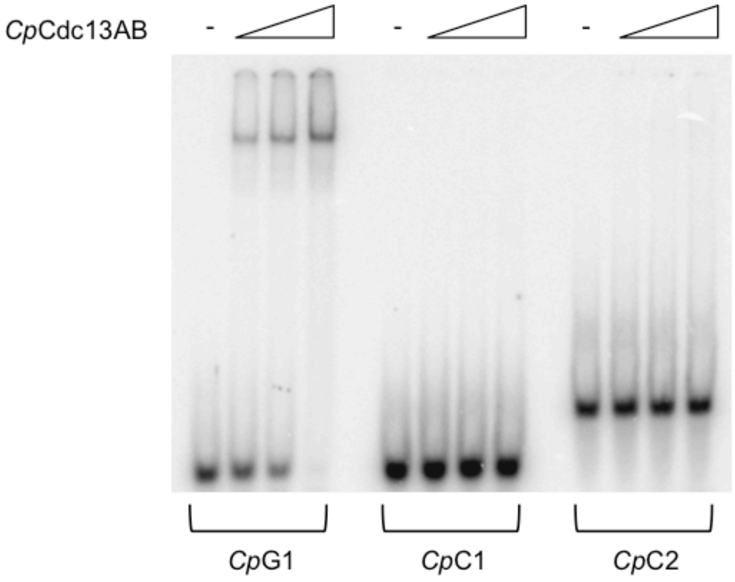

Fig. S3. Comparison of the G-strand and C-strand binding activity of the CpCdc13AB dimer

(Top) The sequences of the oligonucleotide probes used this series of gel mobility shift assaysare listed.

(Bottom) The indicated probes (7.5 nM) were mixed with increasing concentrations of *Cp*Cdc13AB (5, 10, and 20 nM) and complex formation was monitored by native gel electrophoresis.

Fig. S4

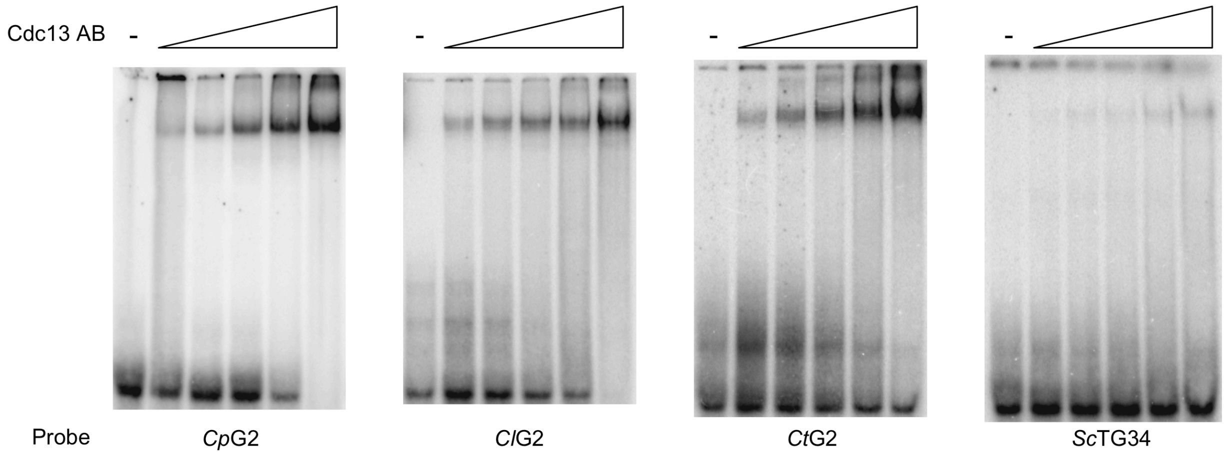

Fig. S4. The binding of the CpCdc13AB complex to the cognate and heterologous telomere oligonucleotide

The interaction between *Cp*Cdc13AB (0, 2.5, 5, 10, 20, 40 nM) and the indicated G-strand oligonucleotides (15 nM) was examined by gel mobility shift assays.

Fig. S5

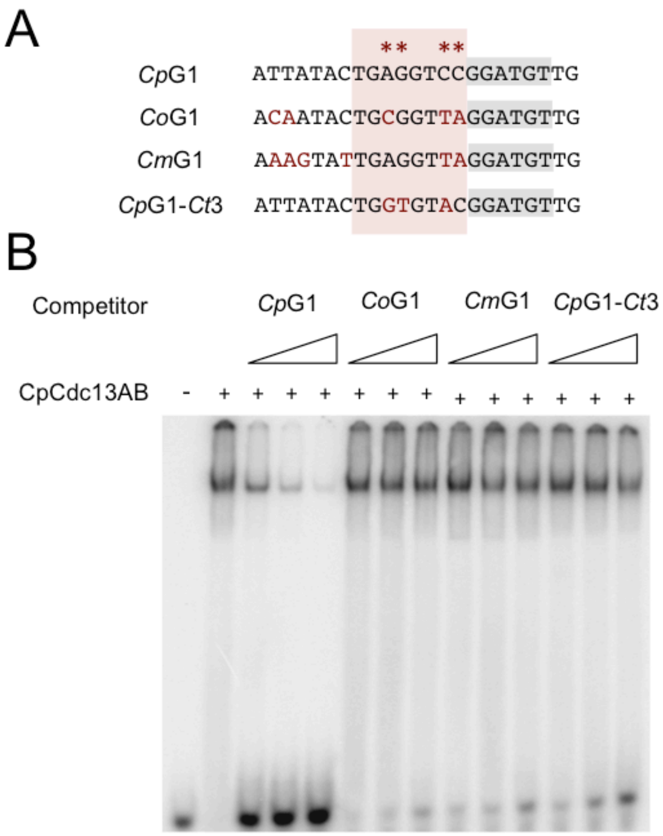

Fig. S5. Binding of CpCdc13AB to heterologous G-strand repeats

(A) The oligonucleotides used in these assays are displayed. *Co*G1 and *Cm*G1 correspond to one G-strand repeat unit of *Candida orthopsilosis* and *Candida metapsilosis* telomeres, respectively. The putative target site for *Cp*Cdc13B is indicated by a light red shaded box, and the positions in the heterologous oligos that differ from the *Cp* G-strand are highlighted in dark red. The four positions that are likely to be recognized by CpCdc13B in a sequence-specific fashion are marked by asterisks.

(B) The interaction between the *Cp*G1 probe (7.5 nM) and the *Cp*Cdc13AB complex (10 nM) was assessed in gel mobility shift assays. Unlabeled *Cp*G1and three variant oligos were added to the assays as competitors. The competitor oligoes were included at 23, 75, and 225 nM.

Fig. S6

A

*Cp*G1	ATTATACTGAGGTCCGGATGTTG
*Cp*G1-*i*1	TACTGAGGTCCAGGATGTTG
*Cp*G1-*i*2	TACTGAGGTCCACACACGGATGTTG
*Cp*G1-*i*3	TACTGAGGTCCACACACACACACGGATGTTG

B

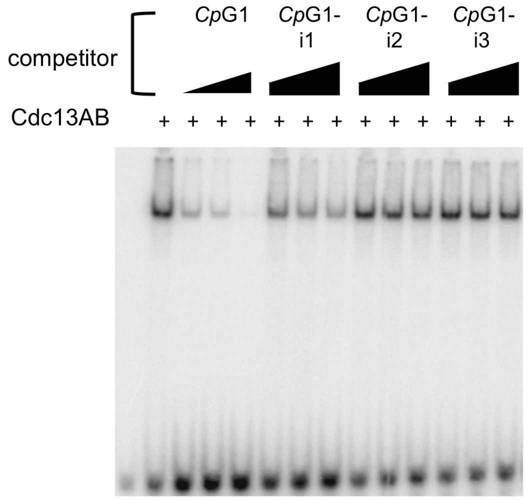

Fig. S6. The effect of nucleotide insertions in the DNA substrate on binding by CpCdc13AB

(A) The insertion oligonucleotides used in these assays are displayed.

(B) The interaction between the *Cp*G1 probe (7.5 nM) and the *Cp*Cdc13AB complex (10 nM) was assessed in gel mobility shift assays. Unlabeled *Cp*G1and three insertion oligoes were added to the assays as competitors. *Cp*G1 was included at 23, 75, and 225 nM, and the insertion oligos were included at 75, 225, and 750 nM.

Fig. S7

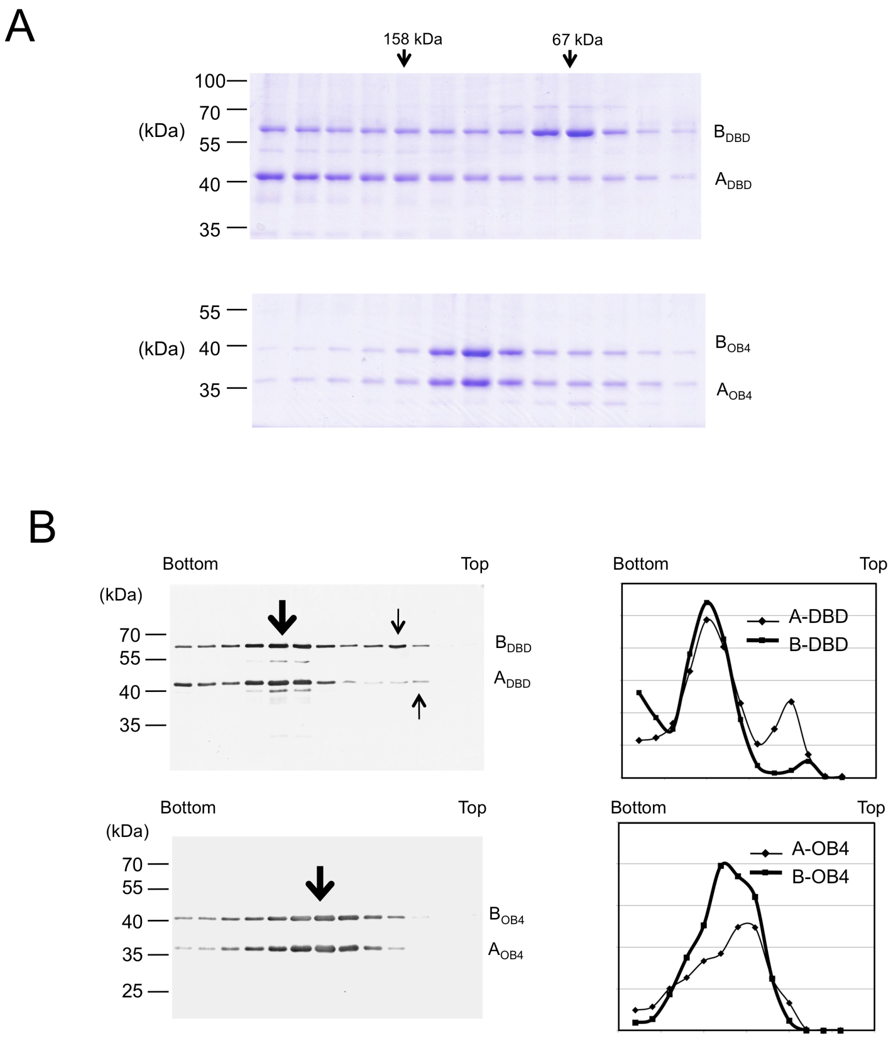

Fig. S7. The sedimentation behaviors of the AB_{DBD} and AB_{OB4} complexes

(A) (Top) The AB_{DBD} complex purified by FLAG affinity chromatography was fractionated through a glycerol gradient. The distributions of A_{DBD} and B_{DBD} in the fractions were analyzed by SDS-PAGE and Coomassie staining. The arrows indicate the positions of the BSA (67 kDa) and aldolase (158 kDa) standards fractionated through a parallel gradient. (Bottom) The AB_{OB4} complex purified by FLAG affinity chromatography was fractionated through a glycerol gradient. The distributions of A_{OB4} and B_{OB4} in the fractions were analyzed by SDS-PAGE and Coomassie staining.

(B) The fractions from the gradients shown in A that correspond to the AB_{DBD} and AB_{OB4} complexes (marked by open arrowheads) were diluted two-fold in Buffer G-0 (50 mM Tris-HCl, pH 7.5, 150 mM NaCl, 2 mM DTT, 0.1 % triton X-100) and subjected to a second round of glycerol gradient fractionation. The distributions of individual subunits were determined by anti-His_6 and anti-FLAG Western analysis, and the results plotted. Large and small arrows in the Western panels are used to indicate the positions of heterodimers and monomers, respectively.
